# The Emergence of Mathematical Understanding: Connecting to the Closest Superordinate and Convertible Concepts

**DOI:** 10.3389/fpsyg.2021.525493

**Published:** 2021-11-22

**Authors:** Zezhong Yang, Xintong Yang, Kai Wang, Yanqing Zhang, Guanggang Pei, Bin Xu

**Affiliations:** ^1^School of Mathematics and Statistics, Shandong Normal University, Jinan, China; ^2^School of Mathematics and Statistics, Northeastern University at Qinhuangdao, Qinhuangdao, China; ^3^School of Foreign Languages, Shandong Normal University, Jinan, China

**Keywords:** mathematical understanding, mathematical concepts, cognitive structure, internal network, connection

## Abstract

This study aimed to examine the specific means and internal processes through which mathematical understanding is achieved by focusing on the process of understanding three new mathematical concepts. For this purpose interviews were conducted with 54 junior high school students. The results revealed that mathematical understanding can be achieved when new concepts are connected to at least two existing concepts within a student’s cognitive structure of. One of these two concepts should be the superordinate concept of the new concept or, more accurately, the superordinate concept that is closest to the new concept. The other concept should be convertible, so that a specific example can be derived by changing or transforming its examples. Moreover, the process of understanding a new concept was found to involve two processes, namely, “going” and “coming.” “Going” refers to the process by which a connection is established between a new concept and its closest superordinate concept. In contrast, “coming” is a process by which a connection is established between an existing convertible concept and a new concept. Therefore the connection leading to understanding should include two types of connections: belonging and transforming. These new findings enrich the literature on mathematical understanding and encourage further exploration. The findings suggest that, in order to help students fully understand new mathematical concepts, teachers should first explain the definition of a given concept to students and subsequently teach them how to create a specific example based on examples of an existing concept.

## Introduction

Mathematical understanding entails knowing, perceiving, comprehending, and making sense of the meaning and connotation of mathematical knowledge. Acquiring mathematical understanding plays an important and crucial role in mathematics learning. Bartlett contended that mathematical understanding can reduce the burden of memory, filter out invalid information in the brain, and maintain the longevity of memory (Bartlett, 1932). Further, Davis observed that it can help students assimilate and transfer knowledge by improving their transferability (Davis, 1992). Pasnak et al. (2016) asserted that it can improve students’ capacity for inductive and deductive reasoning, thereby enhancing their ability to solve mathematical problems fluently. Moreover, Huang and Yu (2002) and Zhang and Wang (2005) emphasized that it can motivate students to acquire additional knowledge (Huang and Yu, 2002; Zhang and Wang, 2005). Lv (2012) argued that by enhancing their ability to solve mathematical problems it can improve students’ ability to solve social problems. Xu (2014) posited that it can improve students’ overall academic performance. Consequently, mathematical understanding has always been a popular topic in the field of mathematics education, and it has attracted the attention of many mathematics education researchers (Hiebert and Carpenter, 1992; Cai and Ding, 2015). In 1989 the National Association of Mathematics Teachers clearly stated that “the focus of the mathematics curriculum should be “mathematical concepts and understanding,” and mathematical education researchers and instructional designers must take mathematical understanding as the primary focus of mathematical research” (Hirsch, 1989). Therefore an exploration of the characteristics of mathematical understanding, especially its internal characteristics, is important and valuable. Accordingly, to extend this line of inquiry, this study aimed to examine the internal processes through which mathematical understanding is achieved in order to enhance mathematics teaching and student learning.

## Literature Review

Because mathematical understanding is very important and valuable, it has been widely researched since the middle of the last century (Pasnak et al., 2016). However, a review of these studies revealed that the literature focused primarily on the overall characteristics (Skemp, 1976; Li, 2009; Bi et al., 2011; Lv, 2013; Wang and Qi, 2014), types (Greeno and Riley, 1987; Zhou, 1998; Zheng, 2001; Wang G. M., 2006; Xu, 2012; Yang, 2012; Lv, 2013), and levels (Buxton, 1978; Herscovics and Bergeron, 1983; Tian, 1993; Pirie and Kieren, 1994; Ma, 2001; Wiggins and McTighe, 2005; Yu and Yang, 2005; Yu, 2006; Xiang, 2007; Martin, 2008; Liu, 2011; Wang and Qi, 2014) of mathematical understanding and the factors that affect it (Perkins and Blythe, 1994; Kong, 2001; Lin and Wen, 2001; Cheng and Huang, 2003; Yuan, 2005; Yu and Yang, 2005; Su, 2006; Yu, 2006; Lei, 2007; Stylianides and Stylianides, 2007; He, 2009; Shi, 2011; Zhang, 2011; Li, 2012; Xu, 2012; Liu, 2015; Zhao, 2016), which correspond the external characteristics of mathematical understanding. Only a few studies have focused on its internal characteristics, i.e., its internal psychological characteristics, especially the internal processes of mathematical understanding.

Reviewing these few studies on the internal characteristics of mathematical understanding, especially on the characteristics of the internal psychological process of mathematical understanding, it can be seen that there are four different views at present. The first view, which is also the earliest one, holds that the internal process of mathematical understanding is one in which mathematical knowledge is comprehended and represented in the learners’ minds and links with each other are established. For example, Lesh et al. (1980) contended that the process of mathematical understanding refers to the state and process in which mathematical knowledge is represented in different ways, and associations between or within these representations are made (Lesh et al., 1980; Post et al., 1982; Wang et al., 2012a).

The second view is that the process of mathematical understanding refers to the transformation of mathematical knowledge representation For instance, Mayer (1989) contended that the process of mathematical understanding involves transmission, reflection, reception, measurement, and transformation. Anderson (2008) conceptualized this in the process of mathematical understanding; an individual changes mathematics knowledge from one representation to another.

The third view believes that the internal process of mathematical understanding is a comprehensive, complex, and iterative process. The most famous scholars who hold this view are Pirie and Kieren (1994). They proposed a theory of mathematical understanding characterized by transcendent recursion. They contended that mathematical understanding is a holistic, dynamic, hierarchical, non-linear, recursive, and internalized psychological process. “Holistic” means that mathematical understanding is a process that involves not only knowledge of mathematics but also knowledge about other domains (e.g., life skills) and practical knowledge. “Dynamic” indicates that mathematical understanding is a process in which many different types of knowledge are integrated. “Hierarchical” suggests that the process of mathematical understanding can be divided into several levels. “Non-linear” implies that mathematical understanding progresses through different routes. “Transcendent recursion” indicates that mathematical understanding is a multi-threaded repetitive process (Pirie and Kieren, 1994; Ma, 2001; Li and Zhang, 2002; Martin, 2008). Additionally, Liu (2009) holds similar views and conceptualizes mathematical understanding as a process that involves the ongoing, dynamic, sublevel, non-linear, and repeated organization and reorganization of knowledge.

The fourth point of view is the most widely held one, which proposes that in the internal process of mathematical understanding, knowledge enters the learner’s brain and interacts with the original knowledge to form a new cognitive structure, which means that it is a cognitive activity. For instance, Davis (1992) contended that in the internal process of mathematical understanding, a new idea is incorporated into a larger framework that has previously existed in the learner’s mind. Zheng (2001) contended that, from a traditional point of view, mathematical understanding refers to the ability to grasp the essence of an object, while from a broader point of view or from the viewpoint of modern psychological perspectives, it is a process of incorporating an object into an appropriate schema. Huang and Yu (2002) conceptualized mathematical understanding as a dynamic process of constructing cognitive structures and assigning meaning to knowledge. Chen and Weng (2003) posited that mathematical understanding involves restructuring, reorganizing, and rebalancing pre-existing cognitions. Wang (2004) believed that mathematical understanding is a cognitive activity that helps individuals gradually understand the essence and laws of mathematics by combining their own knowledge and experience. Yu and Yang (2005) conceptualized that mathematical understanding involves assimilation and adaptation, whereby new mathematical information is incorporated into existing cognitive structures. Zhang and Wang (2005) regarded mathematical understanding as a dynamic process of creating representations and knowledge networks based on existing knowledge. Zhang and Guo (2007) proposed that mathematical understanding is a process by which learners establish links between different domains of knowledge and modify or expand the cognitive structures of their knowledge in these domains. According to Li and Wu (2011), in the process of mathematical understanding, mathematical knowledge enters a cognitive structure and forms an internal network with pre-existing knowledge.

Apparently the above four views are different, although they all address the issue of the internal psychological process of mathematical understanding. The first view emphasizes representation and the connection between representations; the second emphasizes the transformation of representation; the third emphasizes comprehensive regression; and the fourth emphasizes the formation of new cognitive structures. However, they are undoubtedly of great help to our comprehension of mathematical understanding because they help us gain a more in-depth understanding of the internal process of mathematical understanding and shed light on methods for examining mathematical understanding.

Additionally, it is also obvious that these views fail to provide more specific and detailed information about the process of mathematical understanding to help us understand it completely. For instance, the second view believes that mathematical understanding is the process of transformation of mathematical knowledge or its representation, but how is such a representation transformed? What kind of transformation is most conducive to the generation of mathematical understanding? The fourth view insists that in the internal process of mathematical understanding, knowledge “enters” the learner’s brain and interacts with the original knowledge to form a new cognitive structure. But what kind of original knowledge is invoked to interact with the new knowledge? What are the characteristics of the new cognitive structure after the formation of a new mathematical understanding etc.? Due to the existence of such unmapped zones, many mathematics teachers find it difficult to apply these views to practical mathematics teaching (Zhang, 2006; Zhang and Ning, 2006). Therefore it is necessary to undertake an in-depth investigation of this construct to delineate the concrete processes that underlie mathematical understanding and create a detailed profile of the internal characteristics of mathematical understanding. This study contributes to this research area by exploring the internal psychological processes that underlie mathematical understanding. It focuses on the following two research questions:

(a). When new mathematical knowledge is processed, under what specific internal situations does the understanding of it take place?
(b). What kind of previously acquired knowledge present in the cognitive structure is essential for the formation of new mathematical knowledge?

## Theoretical Basis

*Understanding* is a word that educators and researchers often use during the process of teaching and conducting educational research. However, very different perspectives on understanding have been documented in the literature (Cai and Ding, 2015). Greeno (1987) contended that understanding is a method of comprehending a knowledge structure. Chen (1995) observed that understanding is a kind of cognitive activity that involves a search for connections and relationships between things until their essential laws are ascertained. Wiske (1998) conceptualized understanding as the act of transcending available information and creatively using one’s knowledge. Zhu (2004) pointed out that understanding refers to the process of knowing and restructuring experiences to achieve rational control over them.

It is therefore possible that the diverse perspectives on mathematical understanding reflect the various ways in which understanding has been conceptualized. For example, Sierpinska (1987, 1990, 1994) contended that mathematical understanding is an action that helps one understand the meaning of knowledge. According to Simmons (1988), mathematical understanding refers to the unique and profound manner in which individuals perceive, reflect upon, and interpret a subject and express it in different ways. Wang (2006) emphasized that understanding refers to the process in which one uses his/her own experiences and cognitive processes to deal with new things, integrate new knowledge, solve new problems, and, thus, constantly construct and improve his/her own cognitive structure. Zhang and Guo (2007) posited that understanding is a reflection of learning activities, different from memorization and memory. When it comes to the interpretation of the internal process of mathematical understanding, the above-mentioned four viewpoints emerge.

However, most scholars and researchers generally agree that mathematical understanding falls within the purview of mathematics learning. Mathematical understanding is closely related to mathematical cognitive structures and processes. It is the process by which new mathematics knowledge becomes part of an individual’s internal cognitive structure by connecting with previously acquired mathematics knowledge and integrating it with the internal network (Mayer, 1989; Davis, 1992; Huang and Yu, 2002; Yu and Yang, 2005; Zhang and Wang, 2005; Zhang and Guo, 2007; Li and Wu, 2011; Cai and Ding, 2015). In other words, most scholars and researchers generally agree with the fourth view mentioned above. Hiebert and Carpenter (1992) observed that “[a] mathematical idea, procedure, or fact is understood if it is part of an internal network. More specifically, mathematics is understood if its mental representation is part of a network of representations.” In this regard, they made the following observations.

The idea that understanding mathematics makes connections between ideas, facts, or procedures is not new. It is a theme that runs through classic works within mathematics education literature and emerges frequently in more recent discussions of representation and understanding in mathematics. Many of those who study mathematics learning agree that understanding involves recognizing relationships between pieces of information (Hiebert and Carpenter, 1992). In accordance with this perspective, scholars and researchers generally believe that mathematical understanding evolves as representations of mathematical knowledge become interwoven into increasingly structured and cohesive networks. Subsequently, as networks of mental representations of mathematical knowledge grow larger and more organized when new representations are incorporated or new associations are made, one’s understanding is also enhanced (Zhang and Wang, 2005; Li and Wu, 2011). Overall, this growth process is holistic, dynamic, hierarchical, non-linear, transcendent recursive, and an internalized psychological process (Pirie and Kieren, 1994; Ma, 2001; Martin, 2008). During this process an individual will try to represent mathematical concepts in different ways and draw connections between or within representations (Post and Reys, 1979; Post et al., 1982; Anderson, 2008). Therefore the first three views mentioned above are still widely accepted by scholars and researchers.

Many scholars and researchers have contended that as individuals continue to grow and develop, their level of mathematical understanding will transform accordingly. Indeed, “the degree of understanding is determined by the number and strength of the connections. A mathematical idea, procedure, or fact is understood thoroughly if it is linked to existing networks with stronger or more numerous connections” (Hiebert and Carpenter, 1992). For example, Skemp (1976) classified mathematical understanding into two types: instrumental and relational. Instrumental understanding refers to knowledge about what a symbol represents; relational understanding includes not only the knowledge about what symbols represent but also a comprehensive understanding of their nature and relationships (Skemp, 1986). Buxton (1978) divided mathematical understanding into four levels: rote memorization, observation, deep understanding, and logical understanding. Herscovics and Bergeron (1983) divided it into four levels: intuitive, procedural, abstract, and formal. Greeno and Riley (1987) divided it into three types: compliance, implicit, and explicit understanding. Pirie and Kieren (1994) classified it as eight levels: primitive knowing, image making, image having, property noticing, formalizing, observing, structuring, and inventizing (Martin, 2008). Wiggins and McTighe (2005) divided it into five dimensions: explanation, interpretation, application, insight, empathy, and self-awareness. Recently, Yu and Yang (2005) divided it into five levels: zero, common sense, logical, conceptual, and endless. The zero level is characterized by a lack of understanding, which is the beginning of the process of understanding. The common sense level is characterized by rudimentary understanding. The logic level entails deep understanding, which refers to the process of connecting old and new knowledge to form a structure through logical thinking. The conceptual level is a deeper understanding, which refers to the emergence of new concepts on the basis of the formation of new cognitive structures. The endless level is characterized by the acquisition of more meanings or knowledge on the basis of previous understanding after thinking or applying the new knowledge again (Yu and Yang, 2005).

The easiest means to enlarge a network of mental representations is to connect a representation of a new fact or procedure to an existing network. Another method is reorganization, in which [R]epresentations are rearranged, new connections are formed, and old connections may be modified or abandoned. The construction of new relationships may force the reconfiguration of the affected networks. The reorganizations may be local, widespread, and dramatic, reverberating across numerous related networks. Reorganizations are manifested both as new insights, local or global, and as temporary confusion. Ultimately, understanding increases as the reorganization yields more richly connected cohesive networks Hiebert and Carpenter (1992). Existing networks are crucial factors that affect mathematical networks. They affect the relationships that are constructed and their subsequent understanding. Hiebert and Carpenter (1992) observed that “the notion of building understanding by constructing relationships that yield larger, more cohesive internal networks is useful in analyzing a number of issues related to understanding mathematics.”

How are networks of mental representations configured? Current scholars and researchers believe that it consists of many nodes and connections and is very complex. The nodes in this network include elements such as concepts, signs, figures, formulae, axioms, and theorems (Papert, 1993; Wilkerson-Jerde and Wilensky, 2011). The connections are the relationships that exist between nodes. Such networks can be divided into three basic types: linear, tree, and net. A combination of these three basic structures can yield a three-dimensional synthetic structure (Li and Wu, 2011). There are individual differences in the number of nodes and connections, the strength of the connections, and the way in which nodes are connected. The number of nodes and connections has been found to be larger in gifted students, and the distribution of their nodes is uneven (Yang et al., 2018). Regarding the organization of these nodes, many researchers have contended that they can be divided into many different layers (Wo, 2000).

Regarding the relationships that enhance mathematical understanding and those that are formed by the connections drawn between newly and previously acquired mathematics knowledge within an individual’s internal cognitive structure, scholars have contended that they can be classified as two types depending on whether they are based on (a) similarities and differences or (b) inclusion. The former type of relationship is established within a representation form by the noting of the correspondences between different external representational forms and within a given form. They are likely to be found in networks that resemble webs because the delineation of similarities and differences does not necessarily result in the emergence of higher-order relationships. The second type of relationship emerges when one mathematical fact or procedure is perceived to be a special case of another and is based on the notion of inclusion or general and specific cases. Accordingly, such relationships are likely to be found in hierarchical networks (Hiebert and Carpenter, 1992).

In accordance with this perspective, for a long time scholars and educators have always emphasized that, in order to help students understand mathematical concepts appropriately, definitions and specific examples should be presented and explained to students as a part of the teaching process. A definition of a mathematical concept is a statement or description of its connotation and characteristics, and it represents a generic construct that subsumes other lower-order constructs. It indicates the position of a concept within the entire conceptual system, its similarities to other domains, and the differences between them (Cao and Cai, 1989). For example, the following is a definition of a right triangle: a triangle with a right angle is a right triangle. This definition specifies the geometric figure of a right triangle and delineates the difference between a triangle and a right triangle. A specific example of a concept is obviously subsumed by this concept in accordance with its denotation (Cao, 2008). For this reason, many scholars and researchers often examine mathematical understanding by focusing on mathematical concepts as their units of interest (Pirie and Kieren, 1994).

Mathematical understanding is an internal process. Therefore, how can we judge whether an individual has achieved mathematical understanding following mathematical learning, and how can we evaluate his/her degree and level of mathematical understanding? In general, scholars and researchers contend that this can be inferred based on external performance because internal psychological activities always manifest themselves externally (Michell, 1999; Thorndike and Thorndike-Christ, 2009). Additionally, they proposed that an individual’s mathematical understanding should be judged based on his/her comprehensive performance because a single task can be performed correctly even by an individual who lacks adequate understanding. For example, Hiebert and Carpenter proposed that all the following aspects should be assessed to determine an individual’s mathematical understanding: (a) student errors, (b) the relationship between symbols and symbolic programs and corresponding references, (c) the relationship between symbolic procedures and informal problem-solving situations, and (d) the connection between different symbolic systems (Hiebert and Carpenter, 1992).

However, scholars and researchers most commonly use the oral report method to ascertain an individual’s current level of mathematical understanding (Pirie and Kieren, 1994). This method requires students to describe the meaning of their mathematical knowledge in their own words after mathematical learning has occurred; subsequently, experts judge whether their understanding is correct or incorrect (Nickerson, 1985). Evidently, it not only meets the afore-mentioned criteria and operationalizability but is also easier to use than the other methods proposed by Hiebert and Carpenter (1992). For this reason, many scholars and researchers consider it to be an ideal way to assess mathematical understanding accurately (Borgen, 1998; Wang et al., 2012b).

The general criteria for judging mathematical understanding using the oral report method are accuracy and clarity of an individual describing newly acquired knowledge in his/her own words and his/her awareness of the sources of this knowledge. Specifically, if an individual describes the meaning of newly acquired mathematics knowledge in their own words clearly and accurately and can also specify how they acquired this new mathematics knowledge, then they are considered to have understood the respective piece of knowledge. Otherwise, they are considered to have not adequately understood it (Mao et al., 2015). Many existing studies have shown that this is an obvious hallmark of one’s true understanding of knowledge (National Governors Association Center for Best Practices [NGA Center], and Council of Chief State School Officers [CCSSO], 2009). According to Shao (1997), the objective of this method is to ascertain whether an individual is capable of describing a piece of mathematics knowledge in their own words, irrespective of their level of understanding. To ascertain whether an individual has arrived at a deep understanding of a concept, it is necessary to examine whether their narrative of knowledge is detailed, accurate, comprehensive, and systematic (Shao, 1997).

In accordance with the above views and approaches toward mathematical understanding and the practical situation of teaching mathematical understanding, this study adopted the oral report method to assess students’ understanding of mathematical knowledge and hypothesized that: (1) mathematical understanding will be achieved when newly acquired mathematics knowledge is connected to multiple (rather than single) mathematics knowledge acquired previously. The newly acquired mathematics knowledge cannot be understood by merely drawing connections between itself and a single piece of previously acquired knowledge, even though it has entered an existing network and a new network is formed; and (2) one piece of previously acquired mathematics knowledge should be superordinate knowledge of the newly acquired mathematics knowledge. The connections between arbitrary previously acquired mathematics knowledge and newly acquired mathematics knowledge cannot help to realize a complete mathematical understanding.

## Materials and Methods

### Participants

We recruited 54 second-grade students from a junior high school in Jinan, China. Moreover, the academic performance of 14, 27, and 13 students was excellent, average, and poor, respectively. Participant characteristics are summarized in Table 1. Their average age was 13.24 years (SD = 0.4).

**TABLE 1:** Students’ information.

	**Excellent students**	**Average students**	**Poor students**
Male	5	13	8
Female	9	14	5

We recruited second-grade junior high school students because they are older than primary school students and possess foundational mathematics knowledge. Conversely, they are younger than high school students and are yet to acquire substantial amounts of mathematics knowledge. Therefore we speculated that it may be easier and more convenient to teach new mathematics knowledge and examine the extent of their understanding and the underlying processes.

The participants were divided into different groups based on their academic performance. Specifically, they had taken two-semester examinations in the past. The average of the two examination scores was computed and ranked. Using these ranks, they were divided into the following categories: excellent (top 25%), average (between 25 and 75%), and poor (bottom 25%). Because of the simplicity of such an operationalization, this is the most popular means of classifying school students (Maker, 1982; Johnson, 2000; National Council of Teachers of Mathematics, 2000).

This study was conducted in accordance with the recommendations of “The Guidelines of the International Committee of Medical Journal Editors” and “The Adolescent Mental Health Specialized Committee of Chinese Mental Health Association.” Prior to data collection, we obtained written informed consent from all the parents of non-adult participants and all adult participants (i.e., 37 teachers who participated in subsequent interviews). The parents and adult participants provided written informed consent in accordance with the Declaration of Helsinki. This study was approved by the ethics committee of the School of Psychology of Shandong Normal University.

### Instrument

Based on the discussion we had with the students’ mathematics teachers and analysis of previously acquired mathematics knowledge, we chose to focus on the following three mathematical concepts in this study: a twin prime (TP), a hetero-plane straight line (HPSL), and a semi-regular polyhedron (SRP). A TP is a pair of prime numbers with a numerical difference of 2. The HPSL consists of two straight lines in two different planes. The SRP is a convex geometrical figure enclosed by two or more types of polygons. In addition to the afore-mentioned explanations, another reason for focusing on mathematical concepts to study students’ mathematical understanding is that not only are they commonly found in mathematics textbooks for junior high school students, but they also constitute a major proportion of the mathematics knowledge contained in them.

As, according to the mathematics curriculum in China, junior high school students are yet to learn these concepts, we chose to focus on these three concepts. However, our discussions with five junior high school mathematics teachers revealed that it would not be difficult for junior high school students to learn these three concepts because they are closely related to the concepts that they have previously learned.

In order to enhance the brevity and effectiveness of the research instruments, we refined the questions that were used in the study based on the results of a preliminary investigation, in which we conducted interviews with 37 experienced junior high school mathematics teachers (the durations for which they had been teaching mathematics were > 10 years).

The main question that we used in the preliminary investigation was as follows: To help students understand mathematical concepts, what knowledge is it important to teach? Their responses included the names of concepts, their definitions, specific examples, the method of creating examples, graphics, relevant historical knowledge, relevant exercises, and practical applications. These results are consistent with those of Cai and Ding (2015). The details are presented in Table 2.

**TABLE 2:** The frequency of teachers’ answers.

	**D**	**SE**	**MME**	**RHK**	**RE**	**PA**
Frequency	37	36	23	7	14	15
Percentage	100	97.30	62.12	18.92	37.84	40.54

*D, definition; SE, specific examples; MME, method of creating examples; RHK, relevant historical knowledge; RE, relevant exercises; PA, practical application.*

The method of creating examples is a concrete means of generating an example for a new concept based on examples of previous concepts. For example, the following means of deriving an ellipse is a method of creating an example: we can create an oblique section by using a plane to cut a cylinder obliquely. The edges of this oblique section are elliptical; therefore, an ellipse can be derived from a cylinder.

It can be inferred from Table 2 that definitions of concepts, concrete examples, and the method of creating examples were all frequently reported. Therefore we focused on these elements in this study.

For TP, we provided the following method of creating examples: (a) find a prime number and (b) add 2 (larger number) and subtract 2 (smaller number) from this number. If the resultant larger (or smaller) number is also a prime number, then you have identified a pair of TPs. If this larger (or smaller) number is not a prime number, then try another prime number. With regard to the HPSL, we provided the following method of creating examples: (a) first finding a cuboid, (b) drawing two straight lines in their two adjacent planes, and (c) ensuring that they do not intersect on the intersection line of the two adjacent planes and have different angles when compared to the intersection line. The following method of creating examples of an SRP is provided by: (a) finding a cube, (b) connecting the midpoint of each edge, and (c) cutting off eight peripheral triangular pyramids with a plane. The resultant figure is an SRP because it is a convex geometrical figure that is enclosed by two types of polygons (i.e., a regular triangle and square).

### Data Collection

#### Procedure

We defined the three afore-mentioned mathematical concepts and generated three concrete examples and methods for creating concrete examples by interviewing five junior high school mathematics teachers. On four different cards, we wrote down the name of a concept (card A), its definition (card B), a specific example (card C), and a method of creating a specific example (card D). Twelve cards were used.

Individual interviews for collecting information on mathematical understanding were conducted in accordance with the following steps:

(1) One of 54 students was chosen randomly.
(2) One of the three afore-mentioned concepts was selected randomly. The student was shown the card with its name (card A) and asked if they have previously learned about this concept or can understand it. If they had learned about this concept or could understand it, then further exploration was terminated, another concept from the remaining cards was then randomly selected and the student was asked the above question again. If the student could understand all three concepts, the interview was terminated, and we reverted to step (1) choosing another student. This continued until all the students were interviewed. If they did not understand the mathematical concept, only then did we proceed to the next step.
(3) One of the three study plans was randomly selected, namely, α, β, and γ (a detailed description of plans α, β, and γ are presented at the end of this section. Each plan was divided into two parts). The chosen study plan was executed in the following steps:

(a) Implementation of the first part of the plan. When students finished the learning process, they were asked if they had understood the concept. If they said “yes,” they were asked to describe the meaning of this concept in their own words. Then we proceeded to step (b). If they said “no,” then we proceeded directly to step (b).
(b) Implementation of the second part of the plan. When students finished the learning process, they were asked if they have understood the concept. If they said “yes,” they were asked to describe the meaning of this concept in their own words again. If they said “no” again, then the interview that focused on this concept was terminated and we proceeded to step (2) to choose another concept. This continued until all three concepts had been tested.
(c) Students were asked to recount how they transitioned from a state of not understanding the mathematical concept to a state of understanding. They were asked to identify the cards that helped them understand the mathematical concepts, describe the role the contents on that card played in their learning, and how it helped them understand the concept.
(d) Students were asked to identify any other content that helped them understand the concepts. If their responses were valid, they were asked to describe the role played by the specific content and how it helped them understand the concepts.
(e) Students were asked to identify the contents that were unnecessary, and explain why they considered them to be unnecessary.
(f) Students were asked to prioritize the presented content to help other students learn and understand this concept completely.
  After the students finished it, the interview that focused on this concept was terminated.

(4) Step (2) to choose another concept was proceeded to. This continued until all three concepts were tested. During this process, the concepts and plans to be used earlier were abandoned.
(5) Once an interview with a student had been terminated, the interviewer reverted to step (1), chose another student, and repeated the afore-mentioned steps. This continued until all the students had been interviewed.

#### Plans

There are six types of complete permutations for cards B, C, and D. To enhance the efficiency of the research (under the condition of ensuring the effect), we chose three of these permutations according to the results of an advance investigation with 37 experienced junior high school mathematics teachers. They are permutations in BCD, BDC, and CDB. They are considered by most teachers to be helpful for students to understand mathematical concepts compared with the other three permutations. For these three selected permutations, we designed the following plans for the interview:

**Plan α**

Part 1: The student was shown card B, which contained a definition of the mathematical concept, and card C, which presented a specific example of the concept. The student was allowed to read the contents and think about it aloud in order to understand the concept independently.

Part 2: The student was shown card D, which described a method of creating an example of a concept, and allowed the student to read the contents and think about it aloud in order to understand the concept independently.

**Plan β**

Part 1: The student was shown card B, which contained a definition of the mathematical concept, and card D, which described the method of creating an example of the concept. The student was allowed to read the contents and think about it aloud in order to understand the concept independently.

Part 2: The student was shown card C, which presented conceptual examples, and allowed the student to read the contents and think about it aloud in order to understand the concept independently.

**Plan γ**

Part 1: The student was shown card C, which contained a specific example of the concept, and card D, which described a method of creating an example of the concept. The student was allowed to read the contents and think about it aloud in order to understand the concept independently.

Part 2: The student was shown card B, which contained a definition of the mathematical concept. The student was allowed to read the contents and think about it aloud in order to understand the concept independently.

The above procedures and plans are somewhat complicated; however, they can help researchers to identify the specific factors that affect students’ mathematical understanding and explore the internal processes that underlie their mathematical understanding. In view of this, this study firmly adopted them.

Three or four students were interviewed each day after class (i.e., in the afternoons) for a total duration of 3 weeks. Their mathematics teachers determined the order in which they would be interviewed. They were interviewed in a school campus activity room. Four mathematics teachers and four graduate students majoring in mathematics education served as research assistants with their consents. They were required to maintain a detailed and comprehensive record of the selected and used plans and the responses and behaviors of the students.

#### Assessment of Mathematical Understanding

After the interviews were completed, we collected and collated the records of all the research assistants and validated each student’s answers and behaviors. Then we analyzed each student’s description of the concept they had learned in steps (a) and (b) and classified the extent of their understanding of the new concepts. To ensure the objectivity and reliability of the analytic process, we invited two experienced researchers (experts in the assessment of mathematical understanding) to independently analyze the data. Next, the other researchers checked and reviewed their results and discussed the combined results with the two expert researchers. The criteria used to assess students’ mathematical understanding were the accuracy, clarity, and comprehensiveness of their descriptions of the meanings of the newly learned concepts in their own words. If a student’s description of the meaning of a new concept was accurate, clear, and comprehensive, then he/she was considered to have understood the respective concept adequately. If a student’s description of the meaning of a new concept was inaccurate, unclear, or incomprehensive, then he/she was considered to have not understood it adequately. If a student could not describe the meaning of a new concept or his/her description was completely wrong or extremely unclear, then he/she was considered to have not understood it yet. Finally, we examined all the student responses that pertained to the contents they considered important for mathematical understanding and their priorities and analyzed the underlying meanings.

## Results

All the students completed their interviews within 30 min (*M* = 23.13, SD = 6.47). When the names of the new concepts were presented to the students, none of them mentioned that they had seen or heard of them before. This indicated that the three afore-mentioned mathematical concepts were new to the 54 students and were suitable for this study.

### Students’ Understanding of the New Concepts Post-implementation of Part 1 of the Study Plans

Following the implementation of part 1 of plan α, only 12.5 and 17.65% of students had fully understood the new concepts of TP and HPSL, respectively. Most of the students understood the new concepts only partially. Following the implementation of part 1 of plan β, about 70% of students (mainly students with excellent and average academic performance) fully understood the new concepts. Only some students (mainly students with poor academic performance) did not fully understand the concepts, and a few students had not yet understood the concepts. Following the implementation of part 1 of plan γ, only 10.53% of students had fully understood the concepts, and over 71% of students (mainly students with excellent academic performance) partially understood the concepts. In other words, almost none of the students fully understood the new concepts. The students’ understanding of the new concepts following the implementation of part 1 of the plans is summarized in Table 3.

**TABLE 3:** Students’ understanding after part 1 of the study plan.

		**FULL (P)**	**NOT FULL (P)**	**NOT YET (P)**	**Total**
TP	Plan α	2 (12.5)	14 (87.5)	0 (0)	16
	Plan β	17 (70.83)	5 (20.83)	2 (8.3)	24
	Plan γ	0 (0)	12 (85.71)	2 (14.29)	14
HPSL	Plan α	3 (17.65)	14 (82.35)	0 (0)	17
	Plan β	11 (68.75)	5 (31.25)	0 (0)	16
	Plan γ	0 (0)	15 (71.43)	6 (28.57)	21
SRP	Plan α	0 (0)	12 (85.71)	2 (14.29)	14
	Plan β	15 (71.43)	6 (28.57)	0 (0)	21
	Plan γ	2 (10.53)	17 (89.47)	0 (0)	19

*FULL, fully understood; P, percentage, NOT FULL, not fully understood; NOT YET, not yet understood.*

Moreover, group comparisons of their level of understanding post-implementation of part 1 of the three plans for each new concept revealed no significant differences between male and female students and between students with excellent, average, and poor academic performance.

### Students’ Understanding of the New Concepts Post-implementation of Part 2 of the Study Plans

Following the implementation of part 2 of plans α, β, and γ, it could be seen that over 71.43% of the students had fully understood the new concepts. Only a few students (mainly students with poor academic performance) had not yet fully understood the new concepts. The students’ understanding of the new concepts post-implementation of part 2 of the plans is shown in Table 4.

**TABLE 4:** Students’ understanding after part 2 of the study plan.

		**FULL (P)**	**NOT FULL (P)**	**NOT YET (P)**	**Total**
TP	Plan α	13 (81.25)	3 (18.75)	0 (0)	16
	Plan β	20 (83.33)	3 (12.5)	1 (4.17)	24
	Plan γ	10 (71.43)	3 (21.43)	1 (7.14)	14
HPSL	Plan α	14 (82.35)	3 (17.65)	0 (0)	17
	Plan β	14 (87.5)	2 (12.5)	0 (0)	16
	Plan γ	15 (71.43)	4 (19.05)	2 (9.52)	21
SRP	Plan α	13 (92.86)	1 (7.14)	0 (0)	14
	Plan β	18 (85.71)	3 (14.29)	0 (0)	21
	Plan γ	15 (78.95)	4 (21.05)	0 (0)	19

*FULL, fully understood; P, percentage; NOT FULL, not fully understood; NOT YET, not yet understood.*

Additionally, group comparisons of their level of understanding post-implementation of the second part of the three plans for each new concept revealed no significant differences between male and female students and between students with excellent, average, and poor academic performance.

### The Contents That Play the Most Important Role in Understanding New Concepts

The cards that students selected when they were required to identify the contents that played the most important role in helping them understand the new concepts were recorded. After collecting statistics and analysis, we found that all students (including students with excellent, average, and poor academic performance) considered the definitions of new concepts, examples, and the method of creating a specific example, to have played a very important role in helping them understand the new concepts. In particular, about 40% of students believed that definitions and over 31% of students believed that the method of creating a specific example, were important factors that facilitated this process. The statistical results are presented in Table 5.

**TABLE 5:** The most important contents for understanding.

**RC**	**TP**	**HPSL**	**SRP**	**Total**
	**F (P)**	**F (P)**	**F (P)**	**F (P)**
Card B	24 (44.44)	25 (46.3)	21 (38.89)	70 (43.21)
Card C	13 (24.07)	11 (20.37)	12 (22.22)	36 (22.22)
Card D	17 (31.48)	18 (33.33)	21 (38.89)	56 (34.57)

*RC, relevant cards; P, percentage; F, frequency.*

Group comparisons of what the students considered to be the most important content that had helped them understand the new concepts revealed no significant differences between male and female students and between students with excellent, average, and poor academic performance.

### The Prioritization of Contents That Facilitate the Understanding of New Concepts

The responses that students provided when they were required to prioritize the relevant contents that enhanced the understanding of learners, were recorded and analyzed. According to the results, over 50% of the students (including students with excellent, average, and poor academic performance) considered the order “A, B, C, and D” to be the best sequence of presentation of the contents pertaining to new concepts. In other words, they believed that successively presenting the names of concepts, their definitions, specific examples, and the method of creating a specific example would be the most helpful and effective means of helping learners understand a new concept. The detailed results are presented in Table 6.

**TABLE 6:** The best understanding priority.

**RC**	**TP**	**HPSL**	**SRP**	**Total**
	**F (P)**	**F (P)**	**F (P)**	**F (P)**
A, B, C, D	38 (70.37)	28 (51.85)	31 (57.41)	97 (59.88)
A, C, B, D	4 (7.41)	7 (12.96)	5 (9.26)	16 (9.88)
A, C, D, B	4 (7.41)	6 (11.11)	3 (5.56)	13 (8.02)
A, D, B, C	2 (3.7)	6 (11.11)	5 (9.26)	13 (8.02)
C, D, A, B	6 (11.11)	7 (12.96)	10 (18.52)	23 (14.2)

*RC, relevant cards; P, percentage; F, frequency.*

Group differences in the prioritization of the contents that pertained to each new concept revealed no significant differences between male and female students and between students with excellent, average, and poor academic performance.

### The Students’ Oral Reaction During the Implementation of Part 1 of the Plans

In the process of implementing part 1 of plan α, after reading the definition, most students looked at the following examples, and then returned to the description of definition, and started to repeat the keywords in a low voice several times “a pair,” “difference,” “straight line,” “two planes,” “two or more,” and “polyhedrons,” and then said “it should be like this,” “it should be correct,” “that is it.” In the process of implementing part 1 of plan β, after most students had read the definitions and methods continuously, most students turned to the definitions, read them silently again, and then whispered the keywords “a pair,” “different,” “straight line,” “two planes,” “more than two,” and “polygon,” then turned to the method, read it silently again, and then said “this way, this way., understand,” “this way.um., understood.” In the process of implementing part 1 of plan γ, most students went through the examples at first, then turned to the method, read it silently over and over, while whispering “this way. this way. um.,” “somewhat understanding” and “some understanding.”

### Students’ Oral Reaction During the Implementation of the Second Part of the Plans

In the process of implementing part 2 of plan α, after reading the description of the method, most students stared at the description, paused for a while, and then said “Oh, I understand,” “Oh, that’s it. I get it.” At this time, most students raised their heads and looked at the examiner with a smile. In the process of implementing part 2 of plan β, after reading the example, most students said “Well, yes, I understand,” “Yes, no problem. I understand.” In the process of implementing part 2 of plan γ, after reading the definition, most students stared at the definition narrative, and then confidently said, “I understand” At this point, students often looked up and leaned back.

## Discussion

Based on existing studies on mathematical understanding, this study aimed to explore the internal processes underlying mathematical understanding to enhance mathematics teaching and student learning. Using three mathematical concepts as instruments to investigate 54 students, we obtained many results and gained some insights.

### The Important Factors That Affect Mathematical Understanding

The results showed that all students were able to understand the three new concepts following the implementation of parts 1 and 2 of the plans. This finding indicates that the definitions of concepts, use of examples, and the method of creating a specific example enabled students to transition from a state of not understanding to a state of understanding; consequently, these were the important contents that helped students understand the new concepts. This is consistent with the preliminary investigation results and confirms the perspective of Cai and Ding (2015).

A careful analysis of the mathematical understanding of students post-implementation of part 1 of plans α, β, and γ revealed that the definitions of concepts and the method of creating a specific example were not only important but also necessary and indispensable. This is consistent with the findings of some researchers (Cao, 2008; Li and Wu, 2011). Following the implementation of plan α, it could be seen that almost none of the students had fully understood the concept because this plan did not include the presentation of the method of creating a specific example. Following the implementation of plan β, it could be seen that most of the students had fully understood the concept even though this plan did not include the presentation of examples of the new concept. However, it included the method of creating a specific example. Following the implementation of plan γ, it was found that almost none of the students had fully understood the concept because this plan did not include the presentation of the definitions of concepts.

Similar conclusions can be drawn from the contents that the students considered important to their learning and the students’ oral reactions during the implementation of the plans. When they were asked to identify the contents that had played the most important role in helping them understand the new concepts, about 40% of the students named the definitions of the concepts, and over 31% of them named the method of creating a specific example. During the implementation of the plans, when the students learned the definitions of concepts and the method of creating a specific example, most of them said, “I understand,” especially when the definitions of concepts and the method of creating a specific example were arranged occurred after other contents.

### The Role of Definitions and Methods in Mathematical Understanding

As mentioned earlier, the definition of a mathematical concept is a statement or description of its connotations and characteristics. It indicates the position of a concept within the entire conceptual system, the concept that it is similar to, and the differences between them (Cao and Cai, 1989). Accordingly, it reveals the relationship, connection, and difference between a given concept and its superordinate concept (Ausubel et al., 1978).

The same is true for the method of creating a specific example. In this method, an example of a new concept is created based on an example of a known concept. Consequently, the new example also reveals the relationship or connection between a related known concept and a new one, which promotes mathematical understanding. The only difference is that the relationship or connection here, was made through the process of creating a specific example that shows the link between the new concept and the known one (which shares a juxtaposed relationship with the new concept) (Ausubel et al., 1978).

Therefore it is the relationships, connections, and juxtapositions between new concepts and their superordinate concepts that promote mathematical understanding among students. This finding is consistent with previous studies (Mayer, 1989; Davis, 1992; Hiebert and Carpenter, 1992; Huang and Yu, 2002; Yu and Yang, 2005; Zhang and Wang, 2005; Zhang and Guo, 2007; Li and Wu, 2011).

### The Process of Understanding New Mathematical Concepts

If we further analyze the connection between the definitions of mathematical concepts and the method of creating a specific example, we find that they not only made the objects of connection of new concepts different but also altered the direction of its (connection. Definitions of mathematical concepts specify the superordinate concept category to which a new concept belongs; thus the resulting connection delineates the link between the new concepts and their superordinate concepts. When students learn the definition of a mathematical concept, they engage in superordinate learning activities (Ausubel et al., 1978). Consequently, students generally connect new concepts to older ones in their minds. The method of creating an example involves creating an example of an existing concept, from which an example of a new concept can be derived. Thus the resulting connection here is the link between the existing and new concepts. When students learn how to create a specific example, they engage in juxtaposed learning activities (Ausubel et al., 1978). Consequently, students generally relate existing concepts to new ones in their minds.

Therefore the process of understanding new mathematical concepts should be based on the establishment of a connection between new and existing concepts in the minds of learners. This process can be accomplished in two different ways, namely, “going” and “coming.” “Going” refers to the process of connecting a new concept with an existing one whose inclusive level is higher than that of the new concept (which is a superordinate concept). Its connection direction is from a new concept to an existing concept. In contrast, “coming” refers to the process of connecting an existing concept (which is juxtaposed with the new concept) with the new concept. Its connection direction is from the old concept to the new concept.

The students indicated what they considered to be the best sequence of learning mathematical concepts after they had specified the contents that had played an important role in helping them understand the concepts. The results of statistical analyses of their responses revealed that most of the students considered the following sequence to be the most helpful to their process of understanding a new concept: learning the definition of a mathematical concept and then learning the method of creating a specific example. This means that to promote mathematical understanding among students, it may be best to allow them to connect new mathematical concepts to (a) the superordinate concepts and, subsequently, (b) existing juxtaposed concepts. Mathematical understanding is supposed to be a process of “going” proceeding “coming”.

Previous studies have shown that mathematical understanding is a process in which new concepts enter an individual’s cognitive structure, become a part of it, and form a network of relationships with previously acquired knowledge within the cognitive structure (Hiebert and Lefevre, 1986; Davis, 1992; Li and Wu, 2011). In this regard, Hiebert and Carpenter (1992) observed that “the mathematical understanding occurs as representations get connected into increasingly structured and cohesive networks.” However, the present findings reveal that mathematical understanding, especially complete mathematical understanding, does not result from the mere entry of a new concept into the cognitive structure of an individual and its integration within this structure, which is attributable to the connections that are formed between new and arbitrary pieces of previously acquired mathematical knowledge. Instead, connections must be drawn between the new concept and at least two existing concepts within the cognitive structure of an individual. This result supports hypothesis (1) formulated in this study. With regard to the two existing concepts, one should be a superordinate concept, to which the new concept belongs, and the other should be convertible so that a specific example of the new concept can be derived by means of changing or transforming its examples. This result supports hypothesis and (2) formulated in this study.

Additionally, Hiebert and Carpenter (1992) contended that the relationships that result in mathematical understanding primarily include similarities, differences, and inclusion. However, the present findings show that the relationships that result in mathematical understanding should include a new dimension, namely, the dimension of “change” or “transformation.”

## Conclusion

Mathematical understanding plays an important role in promoting student learning and the application of mathematical knowledge. Within the field of mathematics education, research on mathematical understanding has always been a popular topic (Cai and Ding, 2015). Grounded in the existing literature, especially cognitive network theory (Hiebert and Carpenter, 1992), this study focused on three new mathematical concepts to explore the processes that underlie mathematical understanding by using a sample of 54 junior high school students and the oral report method. The results showed that among the many contents that pertained to the mathematical concepts, their definitions, examples, and the method of creating a specific example were considered to be the most important. Notably, the definitions and the method of creating a specific example were considered particularly important.

Based on the contributions of (a) the meaning and role of the definitions of new concepts and (b) the method of creating a specific example of the processes that underlie mathematical understanding, several conclusions can be drawn. First, mathematical understanding is achieved when a connection between new concepts and at least two previously learned concepts is established within the cognitive structure of a learner. One of these two existing concepts should be the superordinate concept of the new concept, or more accurately, the superordinate concept that is most proximal to the new concept. The other concept should be convertible so that a specific example can be derived by changing or transforming its examples. Second, the process of understanding a new concept involves two processes, namely, “going” and “coming.” “Going” is the process in which a connection is established between a new concept and its closest superordinate concept. In contrast, “coming” is a process in which a connection is drawn between an existing convertible concept and a new one. Third, for a new concept to be understood, it should be situated in the middle of a connection between at least two concepts. Finally, in addition to the three dimensions that have been identified by Hiebert and Carpenter (1992), the relationships that result in mathematical understanding should also include a new dimension: change or transformation.

The present findings therefore suggest that in order to help students understand new mathematical concepts, teachers should first present students with the definition and subsequently teach them how to create a specific example based on an example of a previously learned concept. This will facilitate the formation of an interconnected network of new concepts in the minds of students. When teachers explain new concepts to their students, they should emphasize the superordinate concept to which the new concept belongs. Similarly, when teaching students the method of creating an example, they should emphasize that the example can be derived from the example of a previously learned concept.

The present findings (a) delineate the concrete processes that underlie mathematical understanding, (b) clarify the specific ways in which new concepts should connect to existing concepts so that mathematical understanding can be achieved, (c) illustrate the specific form of an internal network following the achievement of mathematical understanding, and (d) enrich the existing literature on mathematical understanding.

Although the mathematical concepts that were selected and examined in this study were all new to the participants, they were all closely linked to the mathematical knowledge that they had previously acquired. Will similar results be obtained if the mathematical concepts that are distant from previously acquired knowledge are presented to students? This is a noteworthy question for further study.

## Data Availability Statement

The datasets generated for this study are available on request to the corresponding author.

## Ethics Statement

Prior to data collection, we obtained the written informed consent of all the parents of non-adult participants and all adult participants (i.e., 37 teachers who participated in subsequent interviews). The parents and adult participants provided written informed consent in accordance with the Declaration of Helsinki. This study was approved by the Ethics Committee of School of Psychology Shandong Normal University.

## Author Contributions

ZY designed the study and checked the data and the manuscript. ZY, KW, and YZ interviewed the students and analyzed the results. ZY and XY wrote the manuscript. GP and BX revised the manuscript and checked the results. All authors contributed to the article and approved the submitted version.

## Conflict of Interest

The authors declare that the research was conducted in the absence of any commercial or financial relationships that could be construed as a potential conflict of interest.

## Publisher’s Note

All claims expressed in this article are solely those of the authors and do not necessarily represent those of their affiliated organizations, or those of the publisher, the editors and the reviewers. Any product that may be evaluated in this article, or claim that may be made by its manufacturer, is not guaranteed or endorsed by the publisher.
